# Dynamic Lysine Acetylation Disrupts Isocitrate Lyase Function and Enables Metabolic Optimisation

**DOI:** 10.1111/1751-7915.70334

**Published:** 2026-03-30

**Authors:** Adrián Martínez‐Vivancos, Beatriz Gomariz‐Turpin, Gema Lozano‐Terol, Rosa Alba Sola‐Martínez, Álvaro Ortega, Julia Gallego‐Jara, Teresa de Diego Puente

**Affiliations:** ^1^ Department of Biochemistry and Molecular Biology (B) and Immunology, Faculty of Chemistry University of Murcia, Campus of Espinardo, Regional Campus of International Excellence “Campus Mare Nostrum” Murcia Spain; ^2^ Biomedical Research Institute of Murcia Pascual, Parrilla–IMIB Murcia Spain; ^3^ Department of Biotechnology and Environmental Protection, Estación Experimental del Zaidín Consejo Superior de Investigaciones Científicas Granada Spain

**Keywords:** genetic code expansion, glyoxylate cycle, isocitrate lyase, lysine acetylation, post‐translational modification

## Abstract

Proteomic studies have suggested that *Escherichia coli* isocitrate lyase (ICL) undergoes multiple acetylation events, partially inhibiting its activity. However, the molecular basis of this regulation and the contribution of individual lysine residues had not been defined. This study demonstrates that acetylation of ICL in *E. coli* is acetyl‐phosphate–dependent and reversible by the CobB deacetylase, establishing a key post‐translational regulatory mechanism within the glyoxylate shunt. Site‐specific acetylation at K13 and K308 inhibits ICL activity by destabilising the tetrameric assembly and rendering the protein more prone to degradation, whereas lysine‐to‐arginine substitutions at these positions alleviate this inhibition, enhancing carbon flux distribution, metabolic flexibility and biomass yield without the burden of plasmid‐based overexpression. Leveraging this regulatory insight, a KR mutant bearing lysine‐to‐arginine substitutions at residues 13 and 308, engineered directly into the chromosomal *aceA* gene, maintained wild‐type growth rates while reducing acetate overflow and improving metabolic balance during glucose depletion and acetate assimilation, leading to a 61% increase in lycopene production. These findings highlight regulatory‐based metabolic engineering as a powerful strategy to optimise bioproduction and pave the way for extending this approach to other central metabolic enzymes to develop robust microbial cell factories for the sustainable synthesis of biofuels, biochemicals and high‐value compounds.

## Graphical abstract

Acetyl‐phosphate–dependent acetylation of *E. coli* isocitrate lyase (ICL) at K13 and K308 inhibits its activity, while CobB‐mediated deacetylation restores function. Introducing K13R/K308R mutations enhances metabolic balance and increases lycopene yield by 61%, providing a promising approach to optimise microbial cell factories through natural regulatory mechanisms.

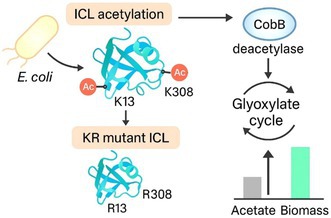

## 1 Introduction

Post‐translational modifications (PTMs) of bacterial proteins play a critical role in regulating diverse cellular processes, including metabolism, transcription, translation, biofilm formation, virulence, stress responses and antibiotic resistance. Among these PTMs, lysine acetylation has emerged as a major regulatory mechanism (Zhang et al. 2009; VanDrisse and Escalante‐Semerena 2019; Luu and Carabetta 2021). Comprehensive acetylome analyses across more than 30 bacterial species have revealed its involvement in essential biological pathways (Popova et al. 2024; Bonifácio et al. 2025). Acetylation dynamics are governed by opposing enzymatic activities: lysine acetyltransferases (KATs) and deacetylases. In addition to these enzyme‐mediated reactions, bacteria also undergo non‐enzymatic acetylation driven by the central metabolite acetyl‐phosphate (AcP) (Christensen et al. 2019). In *Escherichia coli* , both enzymatic and chemical acetylation can be reversed by the NAD^+^‐dependent sirtuin deacylase CobB, the sole known deacetylase in this organism (AbouElfetouh et al. 2015).

Acetylation has been shown to modulate key metabolic pathways, including the glyoxylate shunt (GS), through modification of the central node enzyme isocitrate lyase (ICL) in *Salmonella enterica* (Wang et al. 2010), *E. coli* (Castaño‐Cerezo et al. 2014) and *Mycobacterium tuberculosis* (Bi et al. 2017). However, the molecular mechanism underlying this regulation and the specific lysine residues involved remain poorly understood.

The GS is a bypass of the tricarboxylic acid (TCA) cycle that allows cells to conserve carbon by avoiding oxidative decarboxylation steps. This pathway becomes particularly important when organisms rely on alternative carbon sources such as fatty acids or acetate. In *E. coli* , acetate is consumed once glucose is depleted, a hallmark of carbon overflow metabolism. Redirecting carbon flux through the GS is therefore a promising strategy for improving biotechnological strains engineered for the production of valuable compounds (Causey et al. 2003; Sánchez et al. 2005; Lee et al. 2007; Promubon et al. 2025).

Proteomic studies have identified multiple lysine acetylation sites in *E. coli* ICL, although their functional relevance remains unclear (Castaño‐Cerezo et al. 2014; Schilling et al. 2015, 2019; Christensen et al. 2018; Lozano‐Terol et al. 2024). Based on global acetylome datasets and our MS/MS analyses, lysine residues 13, 193, 308 and 326 were selected for functional characterisation to evaluate their contribution to catalytic regulation. Subsequent experiments focused on K13 and K308, as these sites exhibited dynamic acetylation patterns and exerted the strongest effects on enzymatic activity.

As a proof of concept, we further assessed the biotechnological potential of the K13R/K308R (KR) mutant, which abolishes acetylation‐dependent regulation of ICL at these positions, by evaluating its performance in lycopene production.

Collectively, these findings represent a significant advance in our understanding of the regulatory role of lysine acetylation in central carbon metabolism and provide a framework for the rational design of *E. coli* strains with optimised carbon flux for industrial bioprocessing applications.

## 2 Experimental Procedures

### 2.1 Strains and Plasmids

All strains, plasmids and primers used in this study are listed in Table S1. All molecular biology enzymes were purchased from Thermo Fisher Scientific (Waltham, MA, USA). *E. coli* K‐12 BW25113 *ΔcobB* and *ΔaceA* knockout strains were generated using the phage lambda Red recombination system (Datsenko and Wanner 2000). For ICL overexpression, the *aceA‐ASKA* plasmid was used (Kitagawa et al. 2005). Overexpression plasmids encoding the KR amino acid substitutions of ICL (K13R, K308R and the double mutant K13/308R) were obtained by site‐directed mutagenesis of the *aceA‐ASKA* plasmid. To purify CobB deacetylase, PncA nicotinamidase, YfiQ acetyltransferase, and YiaC acetyltransferase, pRSET overexpression plasmids from laboratory stocks were employed. Site‐specifically acetylated ICL variants at positions K13, K193, K308 and K326 were produced by incorporating N‐(ε)‐acetyl‐lysine via genetic code expansion. A modified pRSF‐Duet‐1 plasmid enabling site‐specific incorporation of acetyl‐L‐lysine (pRSF‐Duet‐1‐acetyl‐lysyl‐tRNA‐synthetase AcKRS3/MbtRNACUA), kindly provided by Prof. Michael Lammers (University of Cologne), was used (de Boor et al. 2015; Lammers 2021). *aceA* genes harbouring amber codons at the target positions were generated by site‐directed mutagenesis, PCR‐amplified and cloned into the pRSF‐Duet‐1‐AcKRS3/MbtRNACUA plasmid, yielding pRSF‐Duet‐aceA‐K13ac, ‐K193ac, ‐K308ac and ‐K326ac constructs.

To engineer an *E. coli* BW25113 strain expressing chromosomal aceA carrying K13R and K308R substitutions, we used the FRUIT method based on *thyA* integration (Rao and Kuzminov 2019). Briefly, the *aceA* locus of a Δ*thyA* mutant (BW25113 Δ*thyA*) was replaced with the wild‐type *thyA* gene using lambda Red recombination. The *aceA* allele containing K13R and K308R was then inserted in place of *thyA*, generating a strain expressing *aceA* with permanent positive charges at positions 13 and 308 and lacking *thyA*.

### 2.2 Protein Overexpression and Purification

Wild‐type *E. coli* BL21 (DE3) cells were made competent using the rubidium chloride method (Hanahan 1983). Competent cells were transformed by heat shock at 42°C with the corresponding overexpression plasmids. Cultures were grown overnight at 30°C with orbital shaking (200 rpm) in lysogeny broth (LB) medium, and protein expression was induced with 0.1 mM isopropyl β‐D‐1‐thiogalactopyranoside (IPTG) at an OD_600_ of 0.5–0.6. For the production of site‐specifically acetylated ICL variants, cultures were supplemented with 10 mM L‐acetyl‐lysine and 20 mM nicotinamide (Sigma‐Aldrich) immediately after induction.

Cells were harvested by centrifugation (20 min, 6000 × g) and resuspended in binding buffer (50 mM potassium phosphate, 500 mM NaCl, 25 mM imidazole, pH 8). Cells were disrupted by sonication on ice (6 cycles of 40 s each) using a 3 mm Vibra Cell VC‐375 probe (Sonics Materials, Danbury, CT, USA) and clarified by centrifugation (30 min, 18,000 × g, 4°C). The supernatant was applied to a 5 mL Ni(II)‐loaded His‐Trap HP column (GE Healthcare) equilibrated with binding buffer. Bound protein was eluted with a linear imidazole gradient (0–500 mM) at 5 mL min^−1^.

The eluted proteins were buffer‐exchanged into conservation buffer (50 mM potassium phosphate, 100 mM NaCl, pH 8) using a HiPrep 26/10 desalting column (GE Healthcare) at 9 mL min^−1^. Purified ICL and its variants were concentrated and analysed by size‐exclusion chromatography on a HiPrep 16/60 Sephacryl S‐300 HR column (GE Healthcare) at 1 mL min^−1^ using a 15–600 kDa calibration kit (Sigma).

### 2.3 In Vitro Acetylation and Deacetylation Assays

Non‐enzymatic in vitro acetylation assays were performed in conservation buffer. ICL (50 μg) was incubated with increasing concentrations of AcP or with 1 mM acetyl‐CoA (AcCoA) at 37°C for 3 h with shaking (200 rpm) in a total volume of 100 μL. For enzymatic acetylation, 10 μg of YfiQ or YiaC and 1 mM AcCoA were added. Control reactions lacking acetyl donors were included.

Deacetylation assays were performed in conservation buffer supplemented with 1 mM NAD^+^. ICL (50 μg) was incubated with 10 μg CobB in a 100 μL reaction. To prevent inhibition by nicotinamide, 10 μg nicotinamidase (PncA) was added. Reactions were incubated at 37°C for 3 h with shaking (200 rpm). Control reactions lacking NAD^+^ were included. Proteins were separated using a Hi‐Trap Q HP anion‐exchange column (GE Healthcare) with a 0.05–1 M NaCl linear gradient at 5 mL min^−1^.

### 2.4 LC–MS/MS Identification of Acetylated Lysines

Acetylated lysines from purified ICL were identified by LC–MS/MS before and after CobB deacetylation and AcP treatment. Full‐length MS analyses followed previously established protocols (Lozano‐Terol et al. 2023). Tryptic digests were analysed using an Agilent 1290 Infinity II HPLC coupled to an Agilent 6550 Q‐TOF mass spectrometer with an AJS‐Dual ESI interface. Data analysis was performed with Spectrum Mill MS Proteomics Workbench (Rev B.06.00.201; Agilent Technologies).

### 2.5 Culture Conditions and Extracellular Metabolite Quantification

*Escherichia coli* BW25113 wild‐type, Δ*aceA*, Δ*cobB* and KR strains were grown in M9 minimal medium supplemented with 20 mM glucose at 28°C with shaking (200 rpm). Cultures were inoculated at an initial OD_600_ of 0.05 from exponential‐phase precultures, and specific growth rates were calculated (Martínez‐Gómez et al. 2012).

To quantify extracellular metabolites (acetate, fumarate, succinate, pyruvate and phosphoenolpyruvate), 1 mL samples were collected at different growth stages, centrifuged (12,000 × g, 1 min, 4°C), and supernatants stored at −20°C. Metabolites were analysed using HPLC equipped with a UV detector and an ion‐exclusion column (ICSep Coregel 87H3, Transgenomic). The mobile phase consisted of 5 mM H_2_SO_4_ at 0.5 mL min^−1^ and 65°C.

Glyoxylate was quantified using a Cosmosil C18‐AR column (Phenomenex, Torrance, USA) at 324 nm with 5% ethanol as the mobile phase in isocratic mode (Long et al. 2023). Metabolite concentrations were calculated from standard curves. Glucose consumption was measured using the dinitrosalicylic acid (DNS) method (Miller 1959).

### 2.6 Isocitrate Lyase Activity

For crude extract activity measurements, 20 mg of *E. coli* cells were collected and centrifuged (10 min, 7000 × g, 4°C). Pellets were resuspended in 50 mM phosphate buffer (pH 7.5) and lysed by sonication on ice (3 × 20 s) using a 3 mm Vibra Cell VC‐375 probe. Lysates were clarified by centrifugation (15 min, 20,000 × g, 4°C) and stored at −80°C. Protein concentration was determined using the bicinchoninic acid (BCA) assay (Pierce BCA Kit; Thermo Fisher Scientific).

ICL activity was measured following (Aoshima et al. 2003). The reaction contained 5 mM MgCl_2_, 20 mM phenylhydrazine and 5 mM DL‐isocitrate. Activity was monitored at 324 nm as glyoxylate reacted with phenylhydrazine (ε = 16.8 M^−1^ cm^−1^). One unit of activity corresponds to the formation of 1 μmol of adduct per minute. Reactions were performed at 37°C in a Synergy H1 microplate spectrophotometer (Bio‐Tek). Activity is expressed as U mg^−1^ protein.

### 2.7 Western Blot Assay

Lysine acetylation levels were assessed by SDS–PAGE (15% acrylamide) followed by semidry transfer to low‐fluorescence PVDF membranes (Trans‐Blot Turbo; Bio‐Rad). Membranes were probed with rabbit monoclonal anti‐acetyl‐lysine antibodies (ImmuneChem) and fluorescent anti‐rabbit secondary antibodies (StarBright Blue 700; Bio‐Rad). Detection was performed with a ChemiDoc MP system (Bio‐Rad). Recombinant enzyme expression was assessed using mouse monoclonal anti‐His_6_ antibodies (Thermo Fisher Scientific) and a StarBright Blue 520 secondary antibody.

### 2.8 Analytical Ultracentrifugation

Sedimentation velocity experiments were carried out on a Beckman Coulter Optima XL‐I ultracentrifuge equipped with an An‐50 Ti rotor. Samples (400 μL) of wild‐type and acetylated ICL variants were prepared at 5, 10 or 15 μM in phosphate buffer (50 mM potassium phosphate, 300 mM NaCl, pH 8). Centrifugation was performed at 42,000 rpm and 20°C, with 150 absorbance scans at 280 nm collected every 7 min. Data were analysed using SEDFIT 17 (Schuck 2000) and visualised using GUSSI (Brautigam 2015). Theoretical s_20_,w values for monomeric, dimeric and tetrameric species were estimated using HYDROPRO (Ortega et al. 2011) and AlphaFold 3‐generated models (Abramson et al. 2024). Solvent viscosity and density were calculated using SEDNTERP (Philo 2023), and partial specific volumes were predicted from amino acid composition.

### 2.9 Lycopene Extraction and Quantification

Lycopene extraction and quantification were performed as described previously (Gallego‐Jara et al. 2015). Extraction was carried out using acetone, and compounds were separated by HPLC with detection at 472 nm. Lycopene concentrations were calculated using response factors relative to the internal standard β‐apo‐8‐carotenal.

### 2.10 Sequence Alignment and Taxonomic Conservation Profiling

To investigate the evolutionary conservation of key lysine residues, we performed a PSI‐BLAST search using the *E. coli* K‐12 ICL (AceA, Uniprot ID: P0A9G6) sequence as a query. The resulting sequence dataset was filtered using CD‐HIT with a 0.90 identity threshold to remove redundancy, and the remaining sequences were aligned with MAFFT using the L‐INS‐i algorithm. The final alignment was loaded into Jalview, where positional conservation was evaluated using the suite of integrated conservation metrics, with residue numbering referenced to the *E. coli* ICL sequence.

To assess conservation across different phylogenetic depths, sequences were additionally grouped into taxonomic categories at multiple ranks. Taxonomic information was retrieved from NCBI Taxonomy, and classification was performed using a custom Python script. For each taxonomic group, sequences were realigned independently with MAFFT, and conservation at the positions of interest was re‐evaluated to identify lineage‐specific trends.

## 3 Results

### 3.1 Acetyl‐Phosphate Drives Reversible Inhibition of Isocitrate Lyase

**FIGURE 1:**
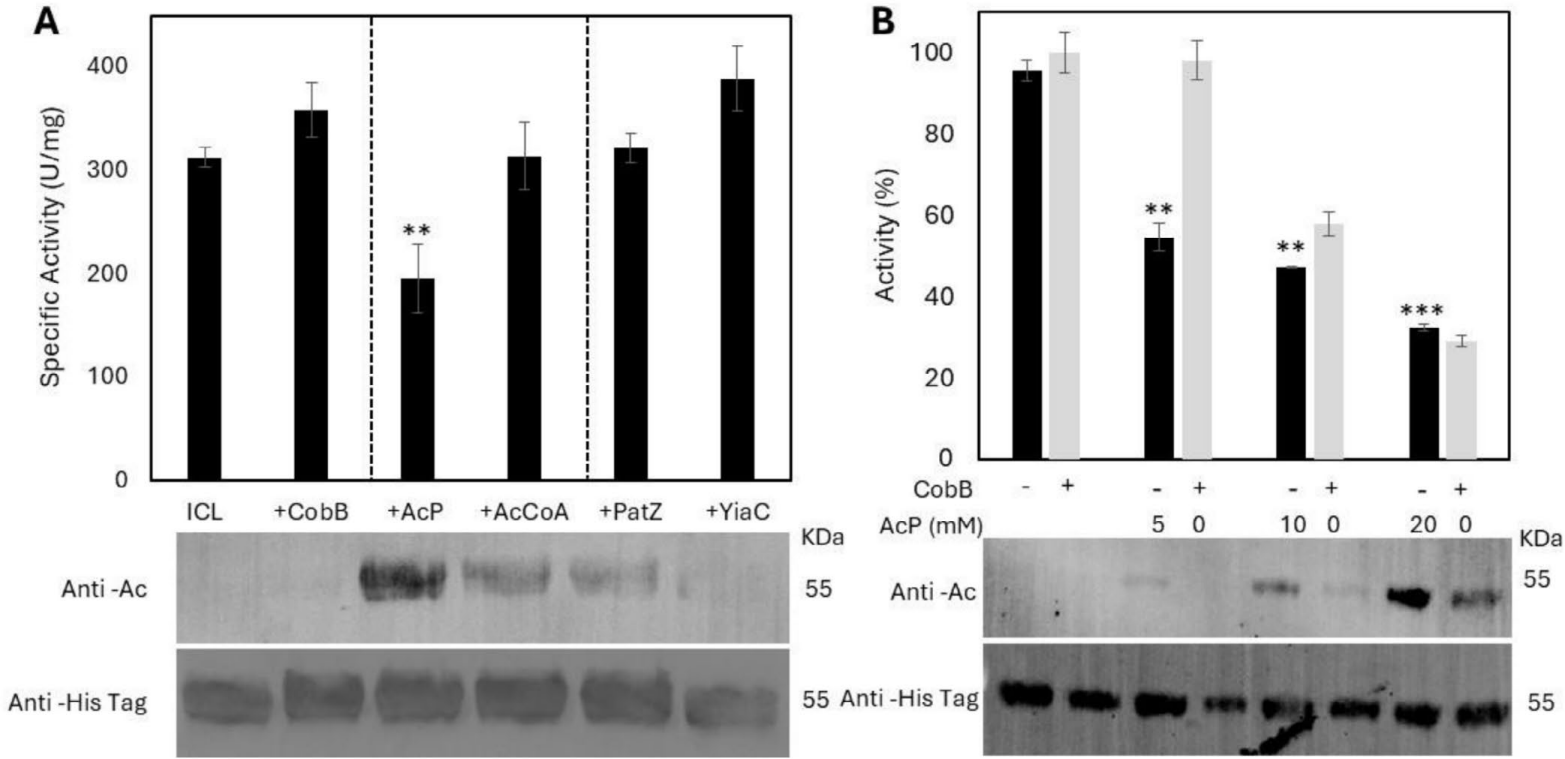
(A) ICL activity on different scenarios. Statistical analysis was performed by Student's *t*‐test comparing each assay with CobB‐deacetylated ICL. (B) Effect of acetyl phosphate (AcP) on ICL activity and reversibility by CobB deacetylation. Western blots probed with anti–acetyl‐lysine and anti‐His antibodies, depicting the degree of chemical acetylation and the relative protein abundance for each assay, are shown at the bottom of both figures. Data are presented as mean ± standard deviation (SD) from at least three independent biological replicates. Statistical analysis was performed by Student's *t*‐test comparing each assay with WT (**p < 0.01; ***p < 0.001).

To determine whether ICL acetylation occurs through a chemical or enzymatic mechanism, we performed a series of in vitro assays. First, we measured the enzymatic activity of freshly purified ICL and of ICL treated with the deacetylase CobB. For chemical acetylation, CobB‐deacetylated ICL was subsequently incubated with the acetyl donors acetyl phosphate (AcP) or acetyl‐CoA (AcCoA). For enzymatic acetylation, the candidate acetyltransferases PatZ/YfiQ and YiaC were tested in the presence of AcCoA. Acetylation in each assay was assessed by Western blotting using an anti‐acetyl‐lysine antibody (Figure 1). ICL expressed and purified from BL21 (DE3) cells showed no detectable acetylation, likely because the enzyme was isolated in a minimally acetylated state. In contrast, acetylation was readily detected after incubation with AcP or AcCoA, as well as in the presence of PatZ/YfiQ or YiaC together with AcCoA. These results indicate that ICL undergoes non‐enzymatic acetylation mediated by both acetyl donors. Activity measurements (Figure 1A) revealed that CobB treatment slightly increased ICL activity, whereas non‐enzymatic acetylation by AcP substantially reduced activity—by approximately 50% relative to CobB‐treated ICL.

We next examined how different levels of chemical acetylation affect ICL enzymatic activity. CobB‐deacetylated ICL was incubated with increasing concentrations of AcP and subsequently subjected to a second CobB‐mediated deacetylation step (Figure 1B). The AcP concentrations tested—5, 10 and 20 mM—were chosen to approximate intracellular levels reported for wild‐type cells and the Δ*ackA* mutant. Consistent with previous observations, immunoblotting confirmed that ICL purified from BL21 (DE3) cells lacked detectable acetylation. As AcP concentration increased, ICL exhibited a progressive loss of enzymatic activity, consistent with the concomitant rise in acetylation levels revealed by western blot analyses. At 5 mM AcP, the enzyme retained only ~50% activity, which was fully restored following CobB treatment, demonstrating the reversibility of acetylation. At higher AcP concentrations, CobB treatment only partially recovered activity. Taken together, these results show that AcP chemically acetylates ICL in a dose‐dependent manner.

To further characterise the acetylation profile of ICL, we analysed the enzyme treated with 20 mM AcP by LC–MS/MS. This analysis confirmed eight lysines previously reported in proteomic studies—K13, K34, K52, K193, K308, K312, K326 and K331 (Castaño‐Cerezo et al. 2014; Schilling et al. 2015, 2019; Christensen et al. 2018; Lozano‐Terol et al. 2024)—and identified seven novel acetylation sites: K62, K63, K82, K173, K212, K381 and K391. Most newly identified residues are surface‐exposed, consistent with the non‐enzymatic nature and low site specificity of AcP‐dependent acetylation (Kuhn et al. 2014). Collectively, these findings expand the known acetylation landscape of ICL.

### 3.2 In Vivo Control of GS Flux by CobB‐Dependent Deacetylation

To investigate the physiological relevance of ICL acetylation, we quantified its enzymatic activity and monitored extracellular glyoxylate and acetate concentrations in *E. coli* wild‐type and Δ*cobB* strains grown in minimal medium containing glucose. Extracellular acetate and glyoxylate were measured as these metabolites diffuse into the medium in proportion to their intracellular production or consumption rates, and their extracellular levels therefore provide an indirect but informative readout of GS activity and carbon overflow. The Δ*cobB* mutant was included because previous studies demonstrated that deletion of *cobB* leads to elevated global lysine acetylation in *E. coli* (Weinert, Schölz, et al. 2013; Castaño‐Cerezo et al. 2014; AbouElfetouh et al. 2015).

**FIGURE 2:**
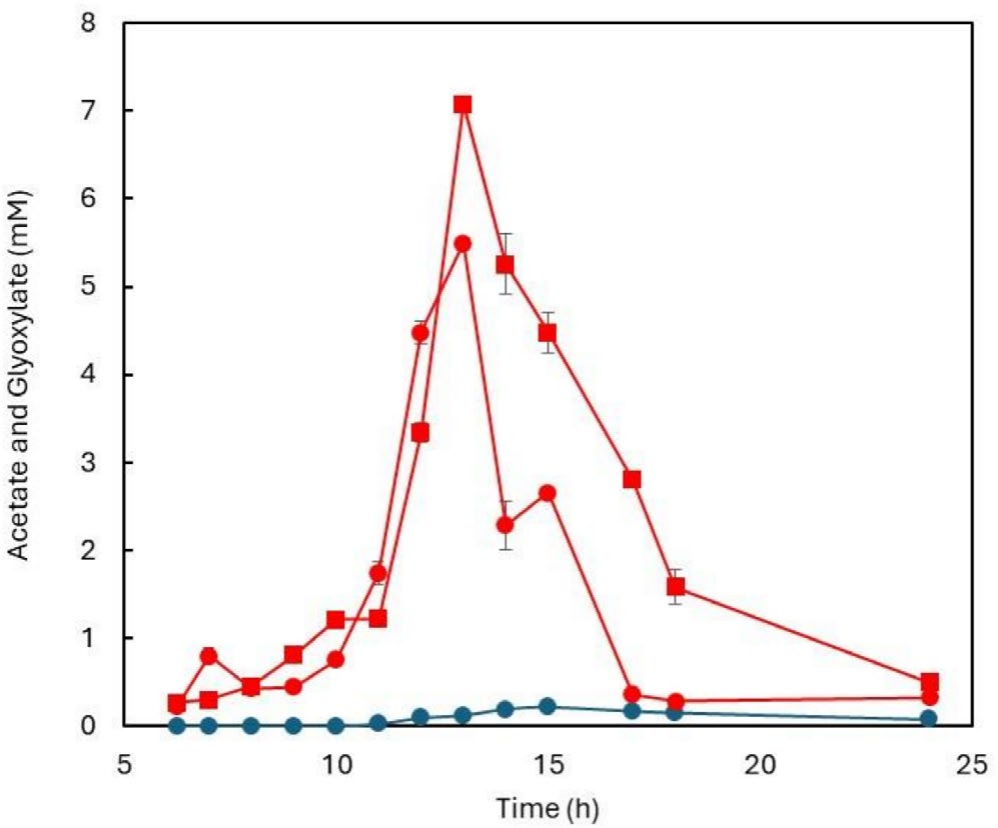
Extracellular concentration of acetate (red lines) and glyoxylate (blue line) in WT (●) and Δ*cobB* (■) strains of *E. coli* .

In wild‐type cells, extracellular glyoxylate concentrations remained low, reaching a maximum of 0.216 ± 0.013 mM at 15 h of cultivation, in agreement with previous reports (Long et al. 2023; Yang et al. 2022). This is expected because glyoxylate is rapidly consumed by malate synthase, thus preventing both its intracellular accumulation and its diffusion into the extracellular milieu (Dolan and Welch 2018; Yang et al. 2022). By contrast, glyoxylate was undetectable in the Δ*cobB* strain, consistent with reduced ICL activity caused by persistent acetylation in the absence of CobB (Figure 2).

‘ICL’ enzymatic activity measured at 12 h (mid‐exponential phase) was 68.5 ± 6.5 mU mg^−1^, with equivalent values independently determined in crude extracts from both the WT and Δ*cobB* strains. From this time point, extracellular acetate started exceeding 3 mM (Figure 2), a threshold at which non‐enzymatic AcP‐dependent acetylation of ICL is likely to start. There is a direct relationship between AcP and acetate concentrations, governed by a dynamic equilibrium dictated by thermodynamic constraints and the kinetic properties of the enzymes involved (Klein et al. 2007; Enjalbert et al. 2017). After 15 h of cultivation, when acetate overflow had reached its maximum, ICL activity in Δ*cobB* extracts decreased 2.6‐fold compared with WT (496 ± 29.7 mU mg^−1^). This reduction is consistent with the enzyme remaining acetylated in the Δ*cobB* mutant, whereas in WT cells CobB rapidly deacetylates ICL under the same conditions. It is known that CobB deacetylase plays a crucial role under metabolic stress in preventing the accumulation of non‐enzymatic acetylation (Weinert, Iesmantavicius, et al. 2013).

The reduced rate of acetate assimilation observed in the Δ*cobB* strain (Figure 2) is consistent with impaired acetyl‐CoA synthetase activity resulting from its hyperacetylation in the absence of CobB‐mediated deacetylation (Castaño‐Cerezo et al. 2014; AbouElfetouh et al. 2015; de Diego Puente et al. 2015; Gallego‐Jara et al. 2017).

### 3.3 Lysine‐to‐Arginine Substitution Enhances ICL Activity by Preventing Acetylation

**FIGURE 3:**
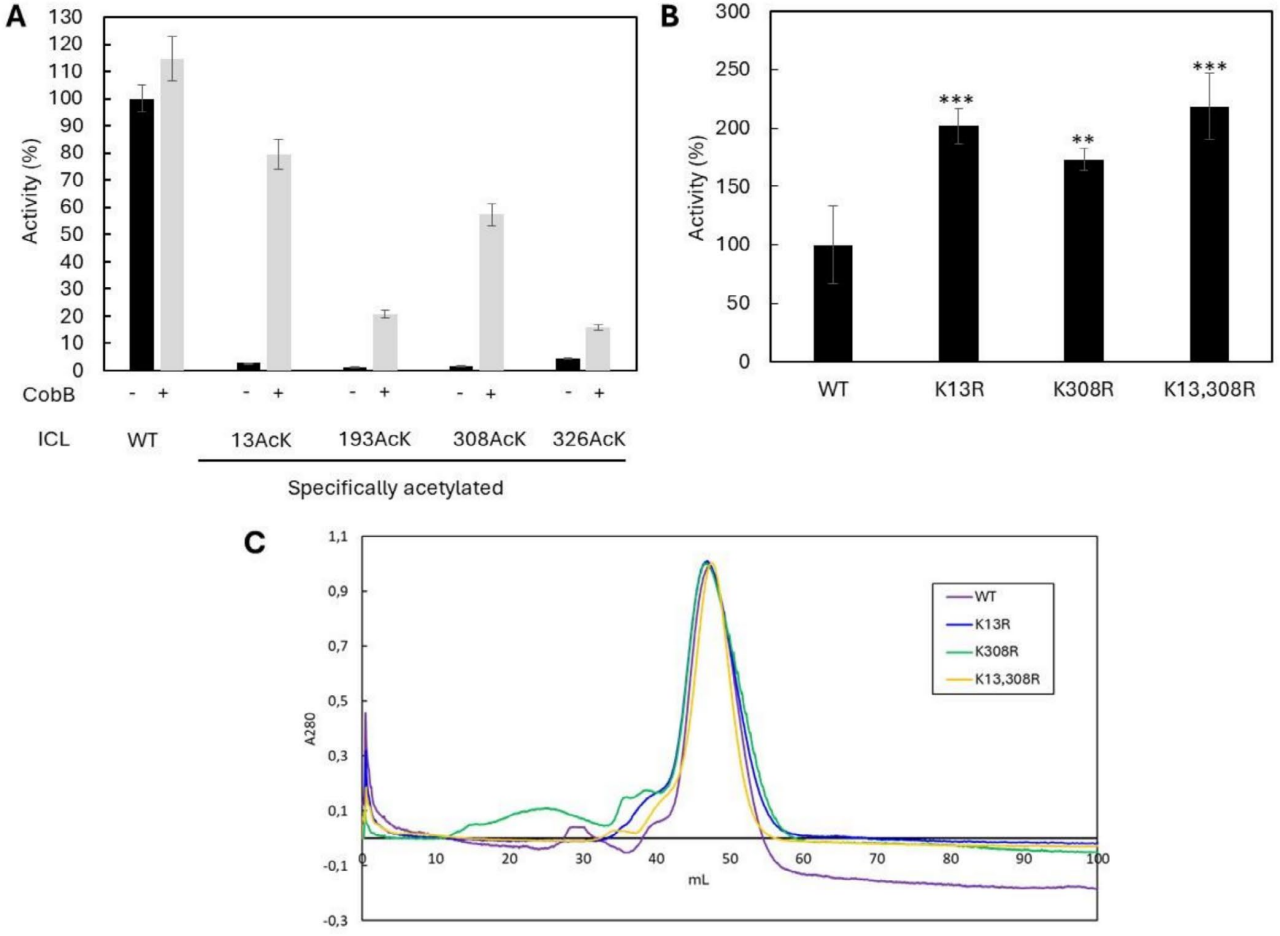
(A) ICL Activity of the WT and specifically acetylated mutants before and after incubation with CobB deacetylase. The data were normalised against the activity of WT protein and plotted as mean ± SD (*n* = 3) and a *p*‐value < 0.001 in all cases. (B) ICL activity of the WT and KR mutants. The data were normalised against the activity of WT protein and plotted as mean ± SD (*n* = 3). Statistical analysis was performed by Student's *t*‐test comparing each mutant protein with WT (**p < 0.01; ***p < 0.001). (C) Size‐exclusion chromatography of WT and KR mutants ICL.

Lysine residues at positions 13, 193, 308 and 326 were selected from those identified by MS/MS in this study for detailed functional characterisation. Proteomic analyses had revealed that these sites undergo differential acetylation under the experimental conditions, suggesting their role as potential regulatory hotspots capable of modulating ICL catalytic activity (Castaño‐Cerezo et al. 2014; Schilling et al. 2015, 2019; Christensen et al. 2018; Lozano‐Terol et al. 2024). Notably, Schilling et al. (2019) reported that acetylation at K13 and K308 responds sensitively to glucose availability and is associated with acetate overflow. Accordingly, these residues were selectively replaced with acetyl‐lysine using a genetic code expansion system. The activity of these site‐specifically acetylated ICL variants was normalised to wild‐type ICL (defined as 100% activity) (Figure 3A).

All acetylated mutants displayed a pronounced reduction in enzymatic activity, retaining < 6% of the wild‐type level. However, after incubation with CobB, only the K13ac and K308ac variants showed substantial recovery, reaching approximately 80% and 60% of the wild‐type activity, respectively. These results indicate that CobB efficiently targets lysines 13 and 308 for deacetylation, supporting their dynamic regulation, whereas residues K193 and K326 do not appear to be physiological CobB substrates. This observation is consistent with previous reports demonstrating CobB‐dependent deacetylation of K13 and K308 (Castaño‐Cerezo et al. 2014; Christensen et al. 2018).

To generate an *E. coli* strain in which ICL activity is not inhibited by acetylation at lysines 13 and 308, we introduced site‐directed mutations replacing these residues with arginine, thereby mimicking a constitutively deacetylated state. Figure 3B shows the enzymatic activities of the resulting mutant variants normalised to wild‐type ICL. Substituting lysine with arginine in the single mutants led to a substantial increase in activity, reaching approximately 2‐fold (K13R) and 1.7‐fold (K308R) relative to the wild‐type enzyme. The double mutant (K13, 308R) exhibited an even greater effect, with a 2.2‐fold increase in activity. Subsequent size‐exclusion chromatography confirmed that the oligomeric state of the KR mutants remained unchanged relative to wild‐type ICL (Figure 3C). These results indicate that preventing acetylation at these positions preserves structural integrity while markedly enhancing catalytic efficiency.

### 3.4 Acetylation at K13 and K308 Destabilises ICL by Disrupting Tetramer Integrity

To further evaluate the impact of lysine acetylation at positions K13 and K308 on the conformational and oligomeric state of ICL, we performed analytical ultracentrifugation sedimentation velocity (SV) experiments on the wild‐type protein and its site‐specifically acetylated variants. ICL is known to adopt a compact tetrameric architecture (PDB ID: 1IGW), and its enzymatic activity critically depends on the formation of stable tetramers, which exhibit enhanced affinity upon ligand binding (Diehl and Mcfadden 1993; Rehman and McFadden 1997; Britton et al. 2001; Grimm et al. 2003). To estimate the expected sedimentation coefficients for different oligomeric species (monomer, dimer, tetramer), we generated the corresponding hydrodynamic models. Three‐dimensional structures of the monomer, dimer and tetramer were obtained using the AlphaFold3 server (Evans et al. 2021; Jumper et al. 2021), and the best‐scoring models were analysed with HYDROPRO (Ortega et al. 2011). Theoretical sedimentation coefficients (s_20_,w) were calculated as 3.5 S, 5.8 S and 9.9 S for the monomer, dimer and tetramer, respectively.

**FIGURE 4:**
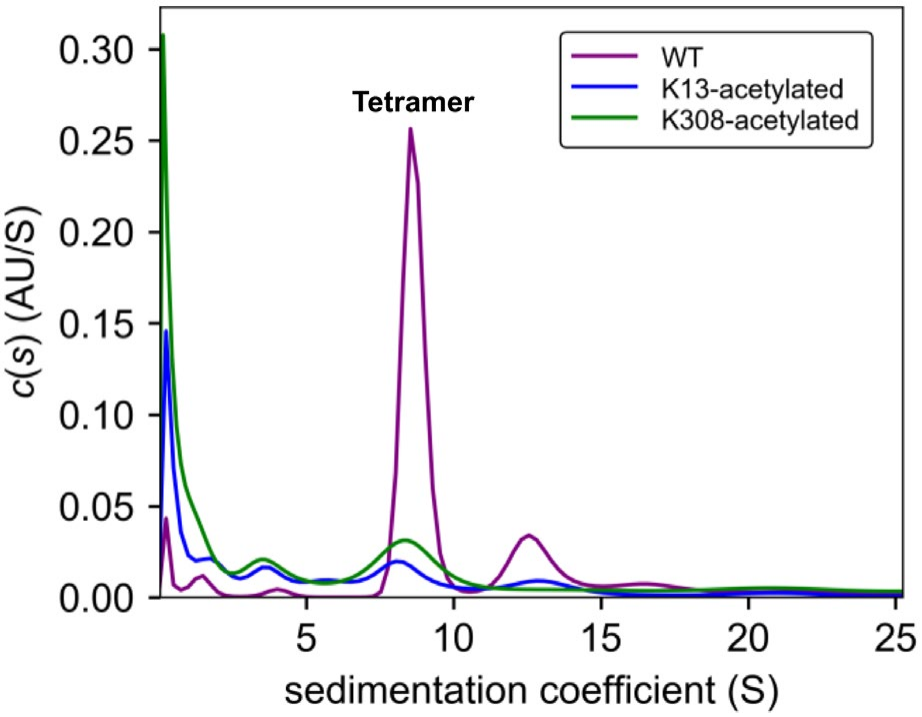
Sedimentation coefficient distributions by analytical ultracentrifugation show the impact of K13/K308 acetylation on ICL oligomerization.

The sedimentation velocity profiles revealed distinct distributions of species between the WT and acetylated proteins (Figure 4). In WT ICL, the predominant species sedimented at 9.6 S, consistent with a tetrameric assembly. Minor species were detected at higher s‐values (14.3 S and 18.6 S), likely corresponding to larger, less stable aggregates. A small fraction at lower s‐values, indicative of partial degradation under the experimental conditions, was also observed. Overall, tetramers accounted for more than 60% of the sedimenting material, aggregates for approximately 30%, and degradation products for < 10%, with negligible monomer or dimer populations.

In contrast, both acetylated variants displayed markedly altered sedimentation profiles. Although the overall distribution of species remained qualitatively similar, the proportions shifted substantially towards smaller oligomers and degradation products (Figure 4). Tetramers represented only ~20% of the total protein in both K13ac and K308ac variants, while monomeric and dimeric species—absent in the WT—accounted for over 20% in K13ac and ~10% in K308ac. Moreover, degradation products exceeded 40% of the total protein in both variants and were accompanied by a reduction in higher‐order aggregates. To rule out non‐ideality effects or concentration‐dependent self‐association, SV experiments were repeated at two higher protein concentrations under identical buffer conditions, yielding consistent results across all dilutions.

Collectively, these findings demonstrate that acetylation at either K13 or K308 severely disrupts the formation of the active tetrameric species and compromises the global structural stability of ICL, rendering the enzyme more susceptible to fragmentation and degradation. The effects of K13 and K308 acetylation were largely similar, although K308ac exhibited a slightly greater tendency towards fragmentation and monomer/dimer accumulation. These observations support a model in which acetylation‐induced tetramer destabilisation contributes directly to ICL inhibition, whereas lysine‐to‐arginine substitutions alleviate this regulatory constraint.

### 3.5 Stable Incorporation of Acetylation‐Free ICL Improves Glyoxylate Cycle Flux

To minimise artefacts associated with plasmid‐based overexpression, we introduced site‐specific mutations directly into the chromosomal copy of *aceA*. This strategy ensures that ICL is expressed within its native regulatory context, preserving physiological expression levels and avoiding the metabolic burden imposed by high‐copy plasmids. By maintaining endogenous expression, this approach enables an accurate assessment of the functional impact of lysine substitutions that prevent acetylation‐mediated regulation, providing insights directly relevant to cellular physiology and biotechnological applications.

**FIGURE 5:**
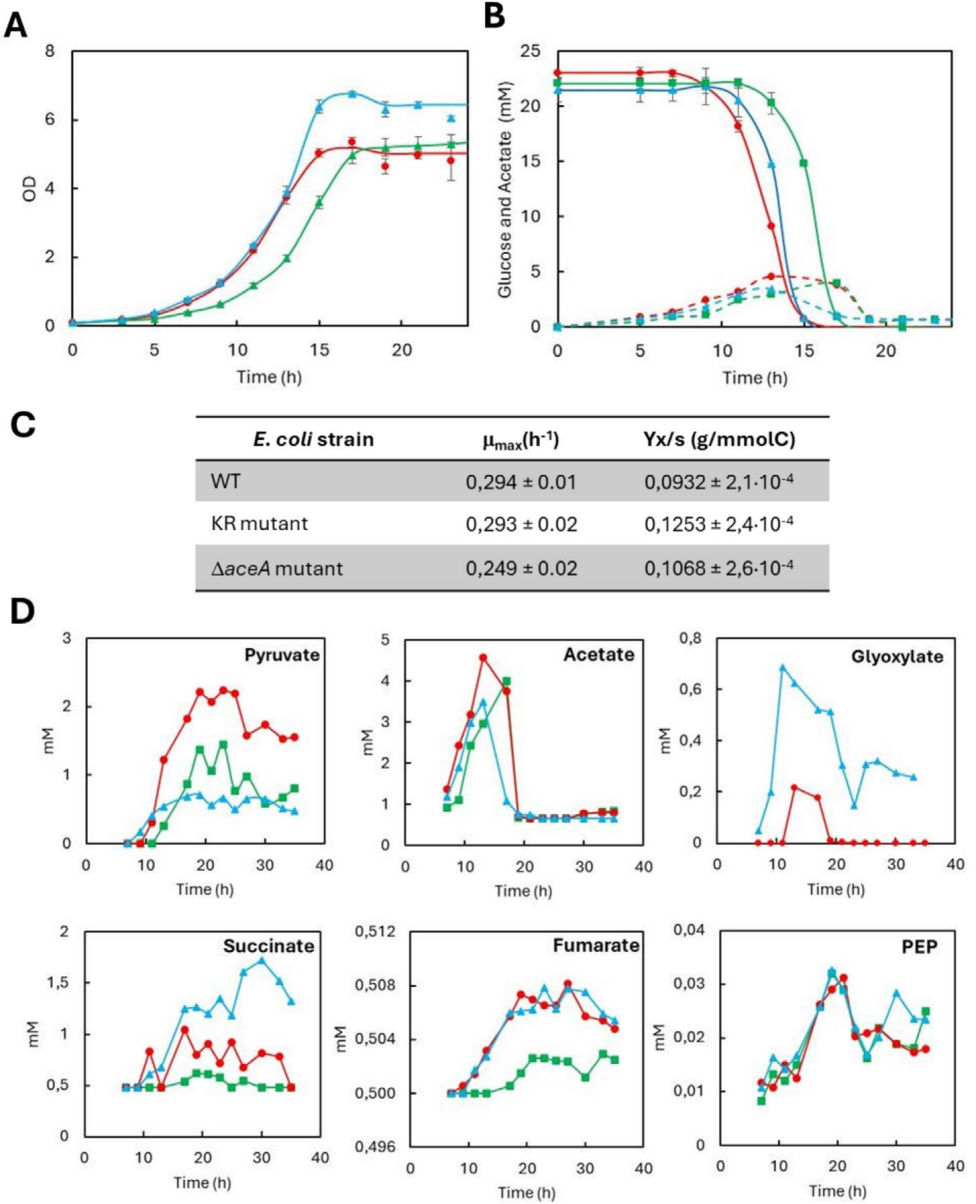
(A) Growth curves of WT (●), KR (▲) and D*aceA* (■) mutants of *E. coli* . (B) Concentration of glucose (continuous line) and extracellular acetate (dashed line) in *E. coli* WT (●) KR (▲) and Δ*aceA* (■) mutants. (C) Specific Growth Rates (μ_max_) and Biomass yield (Yx/s) coefficients for *E. coli* strains. (D) Extracellular metabolite profiles of *E. coli* in WT (●), KR mutant (▲) and Δ*aceA* mutant (■). Data represent the average of three independent experiments with a SD of < 10%.

For this purpose, an *E. coli* strain carrying lysine‐to‐arginine (KR) substitutions at positions 13 and 308 of the chromosomal *aceA* gene was constructed using the FRUIT technique, employing *thyA* as a negative selection marker (Rao and Kuzminov 2019). The physiological characteristics of the KR strain were evaluated by monitoring growth, glucose consumption and extracellular metabolite profiles, using a Δ*aceA* mutant and a wild‐type strain as controls (Figure 5).

The WT and KR strains exhibited the same maximum specific growth rate (μ_max_). However, the KR mutant reached a higher optical density after 15 h of cultivation and achieved a greater biomass yield (Yx/s) than the WT. In contrast, the Δ*aceA* mutant displayed a reduced μ_max_, reaching its maximum OD at 19 h (Figure 5A,C). Both WT *E. coli* and the KR strain fully consumed glucose within 15 h, whereas the Δ*aceA* mutant required 17 h (Figure 5B).

Regarding acetate dynamics, both WT and KR strains reached their maximum extracellular acetate concentration at 13 h, but the KR mutant accumulated a lower peak. Acetate uptake began immediately thereafter and proceeded faster in the KR strain than in WT cells (Figure 5B).

To gain deeper insight into carbon flux distribution, extracellular fermentation products were quantified (Figure 5D). The KR strain exhibited minimal pyruvate accumulation, suggesting a more balanced allocation of flux between glycolysis and the TCA cycle. In contrast, WT and, to a lesser extent, the Δ*aceA* mutant displayed higher pyruvate overflow.

At the isocitrate node, carbon flux is partitioned between the oxidative branch of the TCA cycle and the GS. ICL catalyses the reversible cleavage of D‐threo‐isocitrate into succinate and glyoxylate, a reaction primarily activated during adaptation to growth on acetate (Gui et al. 1996). After 15 h of cultivation, when glucose had been exhausted, both WT and KR strains initiated acetate uptake. At this stage, the KR strain exhibited the highest extracellular glyoxylate concentration (0.623 mM), approximately three times that of the WT, indicating greater ICL activity in the KR background than in the native enzyme. As expected, glyoxylate was undetectable in the Δ*aceA* mutant, since ICL is the sole enzyme responsible for its synthesis in *E. coli* .

Additionally, the KR strain showed higher extracellular succinate levels, which can originate from both the TCA cycle and the GS, whereas succinate in the Δ*aceA* mutant derives exclusively from the TCA cycle. In all strains, extracellular fumarate remained negligible, consistent with its efficient intracellular reutilization. Differences between WT and KR were less pronounced for these metabolites at this stage, although the Δ*aceA* mutant consistently exhibited lower yields.

Finally, phosphoenolpyruvate (PEP), reported as a non‐competitive inhibitor of ICL (Ogawa et al. 2007), displayed similar profiles across all three strains, indicating that the increased ICL activity in the KR mutant is not attributable to differences in PEP levels. PEP leakage was negligible in all cases due to tight intracellular regulation (Escalante et al. 2012).

### 3.6 Site‐Specific Enzyme Modulation as a Strategy for Enhanced Biotechnological Production

**FIGURE 6:**
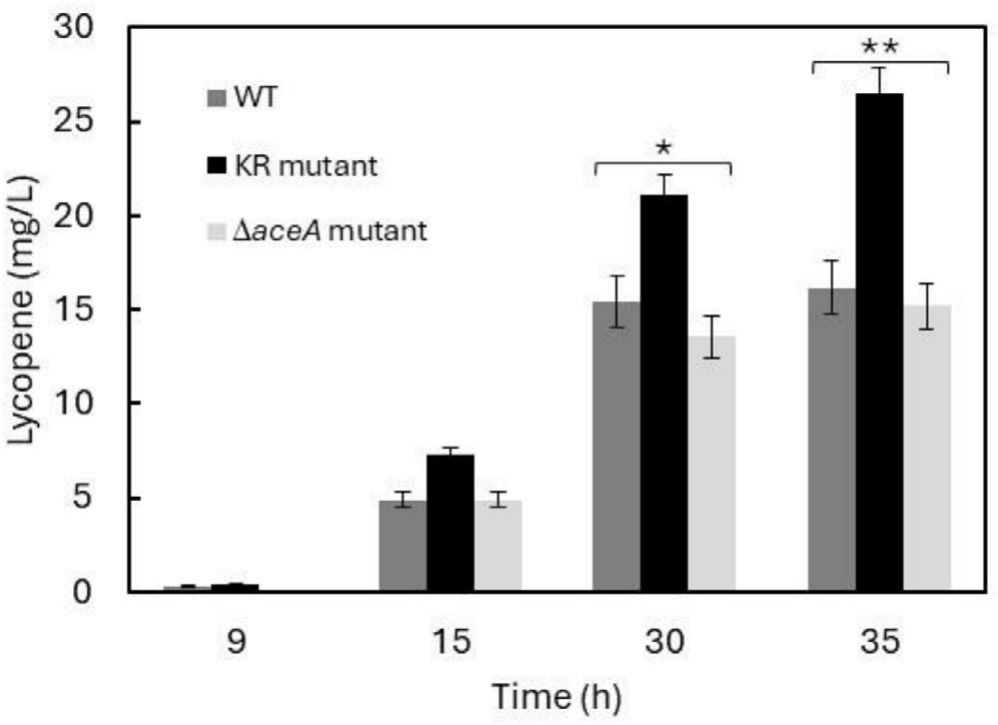
Lycopene production in 50 mL batch culture in MM9 medium containing 20 mM glucose at 28°C. Data represent the means ± SD from three separate experiments. Statistical analysis was performed by Student's *t*‐test, comparing each strain with KR mutant at each time point (*p < 0.05; **p < 0.01).

Building on our previous work on lycopene overproduction (Gallego‐Jara et al. 2015) and considering the diverse metabolic engineering strategies currently available—many of which target regulation of the GS—we assessed lycopene biosynthesis in the three strains under study (Sun et al. 2014; Zhu et al. 2015; Xu et al. 2018; Kim et al. 2021; Gong et al. 2022; Li et al. 2024). The KR and Δ*aceA* mutants, together with the wild‐type *E. coli* strain, were transformed with the pAC‐Lyc plasmid, which carries the three essential genes of the lycopene biosynthetic pathway (*crtE, crtB and crtI*) and subsequently cultivated in minimal medium supplemented with glucose. Intracellular lycopene levels were quantified at different growth stages to characterise production dynamics (Figure 6).

In all strains, lycopene biosynthesis commenced only after glucose depletion, coinciding with an increased flux through the GS. Under these conditions, overflow metabolism was likely alleviated by redirecting gluconeogenic flux towards the synthesis of lycopene precursors. The Δ*aceA* mutant produced lycopene at levels comparable to the wild type, whereas the KR strain achieved approximately 1.6‐fold higher production, highlighting its potential as a promising chassis for biotechnological applications.

## 4 Discussion

In this work, we identified AcP as the main driver of ICL acetylation and inhibition and demonstrated that its effect is reversed by the CobB deacetylase (Figure 1). Although previous acetylome studies indicated that ICL is frequently acetylated under conditions that promote overflow metabolism, the specific regulatory sites and the molecular consequences of these modifications remained unknown. By integrating in vitro reconstitution, site‐specific acetylation, structural biophysics, in vivo physiology and metabolic engineering, we show that AcP‐dependent acetylation at two key lysines—K13 and K308—inhibits ICL activity by destabilisation through compromise of its tetrameric structure. Preventing acetylation at these positions strengthens carbon flux through the GS and substantially enhances lycopene production, demonstrating how post‐translational regulation can be rationally rewired for microbial biotechnology.

These results suggest that non‐enzymatic acetylation plays a major role in metabolic control under conditions that favour elevated AcP levels. Thus, when glucose is depleted and acetate becomes the sole carbon source, CobB‐mediated deacetylation of ICL emerges as a key regulatory mechanism governing carbon flux distribution between the TCA cycle and the GS.

Based on our LC–MS/MS analysis of AcP‐treated ICL, we selected lysine residues 13, 193, 308 and 326, consistent with previous acetylome studies (Castaño‐Cerezo et al. 2014; Schilling et al. 2015, 2019; Christensen et al. 2018; Lozano‐Terol et al. 2024), to evaluate their contribution to catalytic regulation. K13 and K308 emerged as the primary regulatory sites, exhibiting the most pronounced changes in activity and the greatest recovery following CobB treatment, indicating that they are dynamically regulated in vivo. The structural context of these residues explains this behaviour: both lie in solvent‐exposed or flexible regions, making them accessible to CobB, whereas K193 and K326 are more buried and therefore less likely to be enzymatically deacetylated.

Comparative sequence analysis across bacterial taxa reveals that K13 is highly conserved within Gammaproteobacteria class (89%) and Enterobacteriaceae family (97%), supporting a critical functional role. K308 is less conserved globally but remains enriched within Enterobacteriaceae (69%). Notably, approximately 31% of Enterobacteriaceae possess an arginine at the equivalent position, suggesting that maintaining a positive charge at site 308 is essential for structural or functional integrity.

**FIGURE 7:**
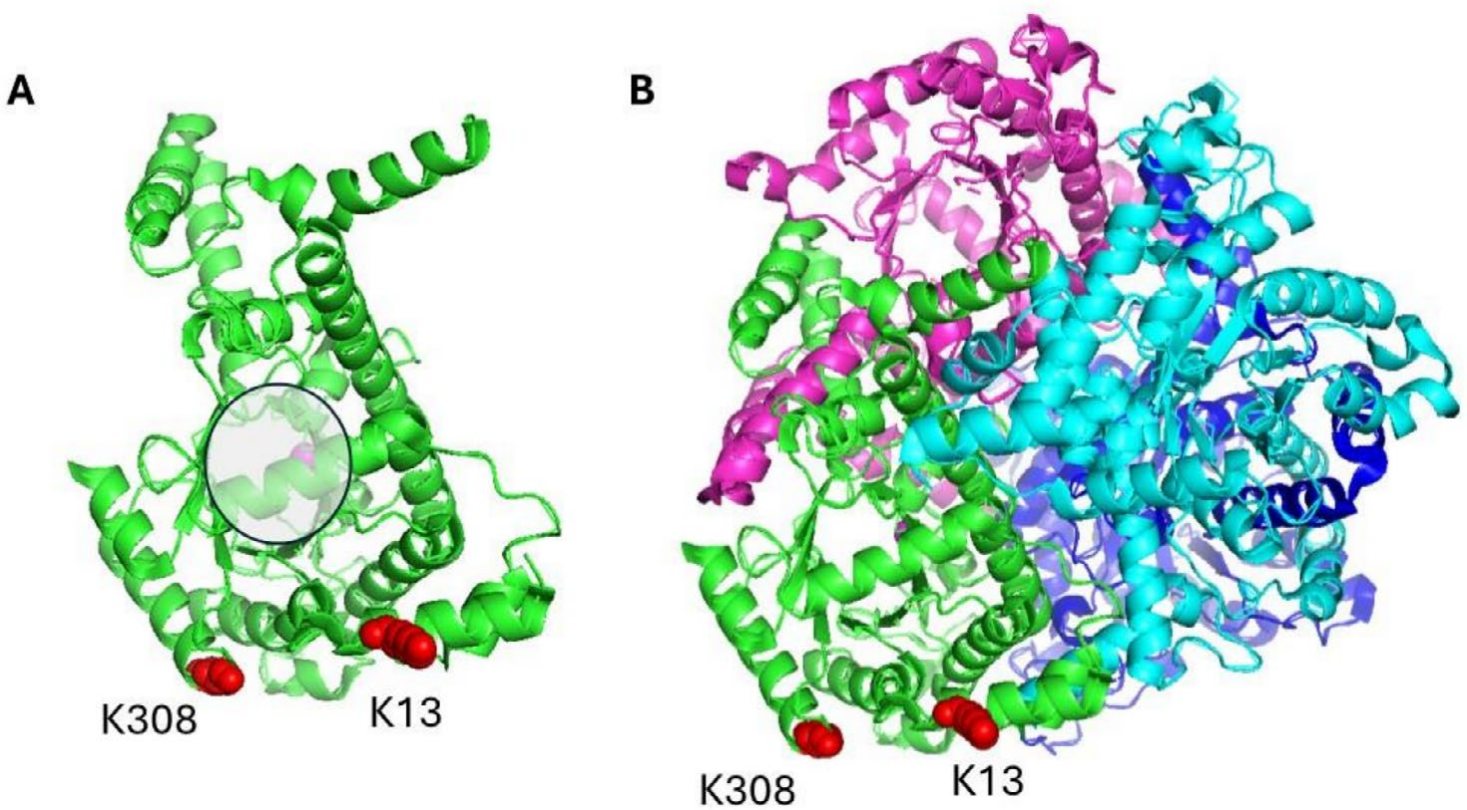
Mapping of key lysine residues of ICL (PDB ID: 1IGW). (A) Monomer. (B) Tetramer. Key lysine residues are labelled in red and monomer active site is marked in grey.

Site‐directed mutagenesis substituting K13 or K308 with arginine led to substantial increases in ICL activity. This enhancement is consistent with the stronger and more versatile electrostatic and hydrogen‐bonding capabilities of the guanidinium group of arginine, which can stabilise local interactions and favour conformations that improve catalytic performance (Sokalingam et al. 2012; Banayan et al. 2024; Dinic and Tirrell 2024). To investigate the structural basis of these regulatory effects, we mapped K13 and K308 onto the crystal structure of ICL (PDB ID: 1IGW) (Figure 7).

Structural inspection revealed that residue 13 resides in an N‐terminal α‐helix (α1), whereas residue 308 lies within a central α‐helical element (α10). Both side chains extend outward towards the solvent, facilitating access by AcP and by CobB (Britton et al. 2001; Weinert, Iesmantavicius, et al. 2013; AbouElfetouh et al. 2015). Mechanisms by which solvent‐exposed residues located far from the catalytic site exert a significant influence on enzyme activity are well documented in the literature (D'Amico et al. 2021; Gu et al. 2023). In most cases, these effects arise from alterations in the dynamics of secondary and tertiary structural elements. However, there are also examples where the primary impact involves perturbations of the quaternary structure (Janin et al. 2008; Marsh and Teichmann 2015). Analytical ultracentrifugation experiments showed that acetylation at either site disrupts tetramer integrity, substantially increasing the proportion of monomeric, dimeric and degraded species (Figure 4). This destabilisation explains the marked loss of ICL activity observed in the acetylated variants. Conversely, KR mutants retain a stable tetrameric architecture, further supporting the link between quaternary structure and catalytic competence.

Engineering an *E. coli* strain carrying chromosomal K13R/K308R substitutions enabled us to evaluate the physiological impact of preventing ICL acetylation in its native regulatory and expression context (Figure 5). Comparative physiological profiling of WT, KR and Δ*aceA* strains revealed that the KR strain exhibits more efficient acetate consumption, reduced overflow metabolism, improved biomass yield and enhanced GS flux. These results demonstrate that bypassing acetylation‐mediated inhibition of ICL increases metabolic flexibility and improves carbon utilisation efficiency when acetate serves as the predominant carbon source.

Although numerous studies have engineered the GS to enhance microbial bioproduction (Yang et al. 2022), our findings show that manipulating regulatory acetylation—rather than expression—can produce equivalent or superior improvements. Using the KR strain as a chassis for lycopene biosynthesis resulted in a 61% increase in production relative to WT (Figure 6). These results reveal that finely tuning post‐translational regulatory layers governing central metabolism can significantly improve metabolic performance in microbial cell factories.

Together, our findings elucidate how lysine acetylation modulates carbon flux distribution and highlight its potential as a target for strain engineering. Removing inhibitory regulatory acetylation at key metabolic enzymes represents a broadly transferable strategy, as many GS, TCA and glycolytic enzymes are acetylated under physiological conditions (Weinert, Schölz, et al. 2013; Popova et al. 2024). This conceptual framework opens new opportunities for redesigning metabolic regulation to improve carbon utilisation, redox balance and precursor supply in engineered microbial systems.

## 5 Conclusion

In summary, we demonstrate, in vitro and in vivo, that acetylation of ICL in *E. coli* is driven by AcP and reversed by CobB, establishing a post‐translational regulatory mechanism that complements transcriptional and metabolic control at the GS/TCA node. Acetylation at K13 and K308 inhibits ICL by destabilising its tetrameric structure, whereas lysine‐to‐arginine substitutions at these positions eliminate this inhibition and enhance carbon flux distribution, biomass yield and bioproduction performance. This strategy led to a 61% increase in lycopene synthesis and illustrates the potential of regulatory‐based metabolic engineering for constructing robust bacterial cell factories. Extending this approach to other acetylation‐sensitive enzymes may enable the development of optimised strains for the sustainable production of biofuels, biochemicals and high‐value compounds.

## Author Contributions

**Adrián Martínez‐Vivancos:** conceptualization, methodology, investigation, formal analysis, writing the original draft. **Beatriz Gomariz‐Turpin:** methodology, investigation, formal analysis, data curation. **Julia Gallego‐Jara:** conceptualization, methodology, investigation, formal analysis, writing – review and editing. **Rosa Alba Sola‐Martínez:** methodology, investigation, formal analysis, writing – review and editing. **Gema Lozano‐Terol:** methodology, writing – review and editing. **Álvaro Ortega:** methodology, investigation, writing – review and editing. **Teresa de Diego Puente:** conceptualization, investigation, formal analysis, writing – review and editing, acquiring the resources, supervision, validation, project administration, funding acquisition.

## Funding

This work was supported by Ministerio de Ciencia e Innovación, PID2021‐122202OB‐I00, JDC2023‐0S1956‐1; Universidad de Murcia.

## Conflicts of Interest

The authors declare no conflicts of interest.

## Supporting information


**Table S1:** Strains, plasmids and primers used in this study.

## Data Availability

The data that support the findings of this study are available from the corresponding author upon reasonable request.
