# Supplementary material for: Dynamic Lysine Acetylation Disrupts Isocitrate Lyase Function and Enables Metabolic Optimisation
Source: Microb Biotechnol. 2026 Mar 30;19(4):e70334. doi: 10.1111/1751-7915.70334 (PMC13140717; doi:10.1111/1751-7915.70334)
Supplement: Supplementary file 1 — Table S1: Strains, plasmids and primers used in this study. [file MBT2-19-e70334-s001.docx]

**Table S1**. Strains, plasmids and primers used in this study.

| **Strains** | | |
| --- | --- | --- |
| BW25113 | *lacI*^+^ *rrnB*_T14_ Δ*lacZ*_WJ16_ *hsdR514*Δ*araBAD*_AH33_ Δ*rhaBAD*_LD78_ *rph1* Δ(*araB–D*)*567* Δ(*rhaD–B*)*568* Δ*lacZ4787*(::*rrnB3*) hsdR514 rph-1 | Invitrogen |
| BW25113 ∆*cobB* | *lacI*^+^ *rrnB*_T14_ Δ*lacZ*_WJ16_ *hsdR514*Δ*araBAD*_AH33_ Δ*rhaBAD*_LD78_ *rph1* Δ(*araB–D*)*567* Δ(*rhaD–B*)*568* Δ*lacZ4787*(::*rrnB3*) hsdR514 rph-1 *cobB* :*kan* | (Gallego-Jara et al., 2017) |
| BW25113 ∆*aceA* | *lacI*^+^ *rrnB*_T14_ Δ*lacZ*_WJ16_ *hsdR514*Δ*araBAD*_AH33_ Δ*rhaBAD*_LD78_ *rph1* Δ(*araB–D*)*567* Δ(*rhaD–B*)*568* Δ*lacZ4787*(::*rrnB3*) hsdR514 rph-1 *aceA* :*kan* | Lab deposit |
| BW25113 KR |  | This study |
| BL21 DE3 | F– *ompT gal dcm lon hsdSB*(*rB- mB*-) λ(DE3). | Promega |
| **Plasmids** | | |
| pKD13 | Amp^R^ /Kam^R^ , resistance cassette flanked by FRT regions. | Yale *E. coli* Genetic Stock Center |
| pKD46 | Amp^R^, this plasmid is temperature sensitive replication at 30°C and arabinose inducible Red recombinase expression. | Yale *E. coli* Genetic Stock Center |
| *aceA*-ASKA | Cam^R^. PT5 promoter. N-terminal, His6-tag overexpression vector, Encodes *aceA* gene. | (Kitagawa et al., 2005) |
| pRSET-yfiQ | Amp^R^. PT7 promoter. Encodes *yfiQ* | Lab deposit |
| pRSET-yiaC | Amp^R^. PT7 promoter. Encodes *yiaC* | Lab deposit |
| pRSET-cobB | Amp^R^. PT7 promoter. Encodes *cobB* | Lab deposit |
| pRSET-pncA | Amp^R^. PT7 promoter. Encodes *pncA* | Lab deposit |
| pRSF-Duet-1- acetyl-lysyl-tRNA-synthetase AcKRS3/MbtRNACUA | Kan^R^. PT7 promoter. Encodes acetyl-lysyl-tRNA-synthetase AcKRS3/MbtRNACUA | (Lammers, 2021) |
| pRSF-Duet aceA K13ac | Kan^R^. PT7 promoter. Encodes acetyl-lysyl-tRNA-synthetase AcKRS3/MbtRNACUA and *aceA* K13 acetylated | This study |
| pRSF-Duet aceA K193ac | Kan^R^. PT7 promoter. Encodes acetyl-lysyl-tRNA-synthetase AcKRS3/MbtRNACUA and *aceA* K13 acetylated | This study |
| pRSF-Duet aceA K308ac | Kan^R^. PT7 promoter. Encodes acetyl-lysyl-tRNA-synthetase AcKRS3/MbtRNACUA and *aceA* K13 acetylated | This study |
| pRSF-Duet aceA K326ac | Kan^R^. PT7 promoter. Encodes *a*cetyl-lysyl-tRNA-synthetase AcKRS3/MbtRNACUA and *aceA* K13 acetylated | This study |
| *aceA*-ASKA K13R | Cam^R^. PT5 promoter. N-terminal, His6-tag overexpression vector, Encodes *aceA* K13R. | This study |
| *aceA*-ASKA K308R | Cam^R^. PT5 promoter. N-terminal, His6-tag overexpression vector, Encodes *aceA* K308R. | This study |
| *aceA*-ASKA K13-308R | Cam^R^. PT5 promoter. N-terminal, His6-tag overexpression vector, Encodes *aceA* K13, 308R. | This study |
| **Primers** | | |
| *aceA*-ASKA K13Ac F | CAACAAATTGAAGAATTACAGTAGGAGTGGACTCAACCGCGTTG | |
| *aceA*-ASKA K13Ac R | CAACGCGGTTGAGTCCACTCCTACTGTAATTCTTCAATTTGTTG | |
| *aceA*-ASKA K193Ac F | GCTGGCGTCAGTGTAGAAATGCGGTC | |
| *aceA*-ASKA K193Ac R | GACCGCATTTCTACACTGACGCCAGC | |
| *aceA*-ASKA K308Ac F | CAAGCTATCCACGCGTAGTATCCGGGCAAACTGC | |
| *aceA*-ASKA K308Ac R | GCAGTTTGCCCGGATACTACGCGTGGATAGCTTG | |
| *aceA*-ASKA K326Ac F | GTCGTTCAACTGGCAGTAGAACCTCGACGACAAAAC | |
| *aceA*-ASKA K326Ac R | GTTTTGTCGTCGAGGTTCTACTGCCAGTTGAACGAC | |
| *aceA* pRSF-DUET F | GTTGTTGAATTCatgAAAACCCGTACACAACAAAT | |
| *aceA* pRSF-DUET R | GTTGTTGGTACCttaGAACTGCGATTCTTCAGTG | |
| *aceA*-ASKA K13R F | CAAATTGAAGAATTACAGCGAGAGTGGACTCAACCGC | |
| *aceA*-ASKA K13R R | GCGGTTGAGTCCACTCTCGCTGTAATTCTTCAATTTG | |
| *aceA*-ASKA K308R F | CAAGCTATCCACGCGCGATATCCGGGCAAAC | |
| *aceA*-ASKA K308R R | GTTTGCCCGGATATCGCGCGTGGATAGCTTG | |
| BW25113 ∆thyA F | TGGTCTGGGCATATCGTCGCAGCCCACAGCAACACGTTTCCTGAGGAACCatgATTCCGGGGATCCGTCGACC | |
| BW25113 ∆thyA R | GGCGTCGGCTCTGGCAGGATGTTTCGTAAttaGATAGCCACCGGCGCTTTTGTAGGCTGGAGCTGCTTCG | |
| BW25113 aceA:*thyA* F | TACCGCCTGTTAGCGTAAACCACCACATAACTATGGAGCATCTGCACatgAAACAGTATTTAGAACTGATG | |
| BW25113 aceA:*thyA* R | GGCCTACAGTCAGCAACGGTTGTTGTTGCttaGAACTGCGATTCTTCAGTttaGATAGCCACCGGCGCT | |
| BW25113 *thyA*:*aceAK13_308R* F | GGCTACCGCCTGTTAGCGTAAACCACCACATAACTATGGAGCATCTGCACatgAAAACCCGTACACAACAA | |
| BW25113 *thyA*:*aceAK13_308R R* | TGCGGCGTGAACGCCTTATCCGGCCTACAGTCAGCAACGGTTGTTGTTGCttaGAACTGCGATTCTTCAGT | |
